# Supplementary material for: Evolving phenotypes of non-hospitalized patients that indicate long COVID
Source: BMC Med. 2021 Sep 27;19:249. doi: 10.1186/s12916-021-02115-0 (PMC8474909; doi:10.1186/s12916-021-02115-0)
Supplement: Supplementary file 1 — Additional file 1: Figure S1. Schema for counting diagnosis records in the cases and controls. Figure S2. Patient population selection. Table S1. Demographic characteristics of the study cohort. Table S2. Manual chart review of the 42 phenotypes identified by MHLO. Table S3. Multivariate ORs for PASC phenotypes [53–60]. [file 12916_2021_2115_MOESM1_ESM.docx]

**Additional File 1**

**Evolving Phenotypes of non-hospitalized Patients that Indicate Long Covid**

**eMethods**

We limited the diagnosis data, by only using the first observation of the records (to minimize the problem list repetitions) and only considered the diagnosis records that for the first time appeared in a patient’s medical records two months or longer after the RT-PCR test (Figure 1S).

The list and description of ICD-9/10 code mapping to Phecodes are described in [53, 54].


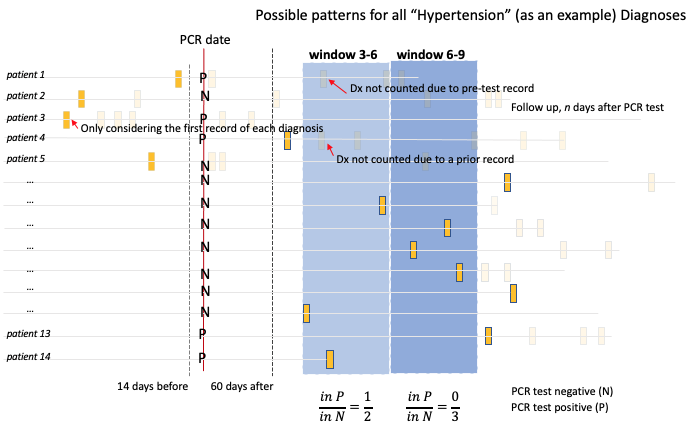


Phenotypes were selected only if the first presentation of their respective ICD code in the patients’ medical record since 2010 was 60 days or longer after the RT-PCR test.

**Figure S1.** Schema for counting diagnosis records in the cases and controls.

**MLHO phenotype selection criteria**

In an iterative process in which the data is iteratively (100 times) split into train and test sets with a 75-25% ratio) the phenotypes in the train sets are first fed to Minimize Sparsity, Maximize Relevance (MSMR)[[23, 32, 33]](https://paperpile.com/c/vO48re/meVZK+acT3+aRT0) algorithm. MSMR first applies a sparsity screen, removing phenotypes that were observed in smaller than 0.2 percent$(\frac{1}{500})$of the patients. Second, MSMR computes the Joint Mutual Information (JMI).[[55, 56]](https://paperpile.com/c/vO48re/HJmB0+uSQIP) The JMI score provides a nonlinear entropy-based criteria for feature selection that also takes into account the redundancy between the features: two features could each be highly relevant on their own, but also be strongly correlated. The MSMR algorithm enables for a wide search of phenotypes in a large feature space. MSMR narrowed down the list of phenotypes to 25 percent.

Using the remaining phenotypes, MLHO trains multivariate Generalized Linear Model (GLM) with component-wise functional gradient boosting models with a logit link and binomial distribution. The boosting algorithms improve the prediction power of the model by training a sequence of weak models that each compensate for the weaknesses of their predecessor, encouraging sparse solutions.[[57-60]](https://paperpile.com/c/vO48re/qfxeC+chzHq+RfRde+qTid8) The GLM boosting algorithm provides an additional multivariate layer for feature selection. We extracted the regression coefficients and identified the phenotypes that are consistently selected through boosting and positively associated with a COVID-positive test result in the past. To do so, we compute a composite score based on the number of times a phenotype was identified in the overall 100 iterations as well as the odds ratios from the GLM model. If a phenotype is identified in all runs with a positive association, it will get a score of 100. If in some of the iterations the association turns negative, MLHO considers this as potential noise and applies a 100 percent penalty. That is, if a phenotype is identified as positively associated with a past positive PCR test in 50 iterations, but negatively associated in 10 iterations, the confidence score will be $50-(10\times2) =30$. No such incidence was recorded. We selected phenotypes based on a composite score of higher than 80 to present in this study. The composite score represents our confidence in the identification of a phenotype at a given temporal window.


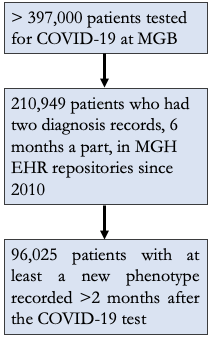


From nearly 400 thousand patients who had tested for COVID-19 at an MGB facility, over than 96 thousand met the inclusion criteria for the study.

**Figure S2.** Patient population selection.

**Table S1.** Demographic characteristics of the study cohort

|  |  | COVID-19 positive test rate |
| --- | --- | --- |
| White | 73,281 (76.3%) | 20.2% |
| Black of African American | 7,246 (7.5%) | 31.8% |
| Asian | 3,012 (3.1%) | 17.7% |
| Hispanic | 3,451 (3.5%) | 43.3% |
| non-Hispanic | 92,572 (96.4%) | 22.6% |
| Female | 61,409 (63.9%) | 23.3% |
| Male | 34,615 (36%) | 23.5% |
| Age | Mean: 50.7 yrs  Mean Covid-negative: 51.5 yrs  Mean Covid-positive: 48 yrs | # novel phenotypes  3-6 months: 1,611  6-9 months: 1,401 |

The study cohort included representation from a diverse population, with an average age of nearly 51 years old. Novel phenotypes identified before screening for sparsity were observed in over 0.22% of patients.

**Table S3.** Manual chart review of the 42 phenotypes identified by MHLO.

| **Phenotype** | **Phenotype verified in clinical note (no. of charts verified / no. of charts reviewed)** | **Phenotype verified as a new symptom (no. of charts verified / no. of charts reviewed)** |
| --- | --- | --- |
| Acute cystitis | 5/5 | 5/5 |
| Alopecia | 5/5 | 5/5 |
| Anemia during pregnancy | 5/5 | 5/5 |
| Anemia of chronic disease | 3/5 | 3/5 |
| Anosmia and dysgeusia | 5/5 | 5/5 |
| Cholelithiasis | 4/5 | 1/5 |
| Chronic fatigue syndrome | 4/5 | 4/5 |
| Chronic kidney disease, Stage I or II | 4/5 | 2/5 |
| Conjunctivitis, infectious | 5/5 | 5/5 |
| Dementias | 5/5 | 3/5 |
| Diseases of nail, NOS | 5/5 | 4/5 |
| Disorders of conjunctiva | 5/5 | 5/5 |
| Dizziness and Light-headedness | 4/5 | 4/5 |
| Heart failure with preserved EF | 5/5 | 5/5 |
| Hypertensive heart disease | 5/5 | 3/5 |
| Impaired fasting glucose | 4/5 | 2/5 |
| Iron def. anemias, unspecified | 5/5 | 5/5 |
| Irregular menstrual cycle | 4/5 | 4/5 |
| Disaccharide intolerance | 5/5 | 0/5 |
| Left bundle branch block | 5/5 | 1/5 |
| Major depressive disorder | 4/5 | 3/5 |
| Malposition and malpresentation of fetus or obstruction | 4/5 | 4/5 |
| Nonrheumatic aortic valve disorders | 1/5 | 1/5 |
| Nonspecific chest pain | 5/5 | 4/5 |
| Open-angle glaucoma | 2/5 | 1/5 |
| Other abnormality of urination | 3/5 | 1/5 |
| Pain, swelling or discharge of eye | 4/5 | 4/5 |
| Palpitations | 5/5 | 5/5 |
| Peripheral or central vertigo | 5/5 | 5/5 |
| Pneumonia | 4/5 | 4/5 |
| Primary open angle glaucoma | 4/5 | 1/5 |
| Proteinuria | 5/5 | 4/5 |
| Rash and other nonspecific skin eruption | 5/5 | 5/5 |
| Sensorineural hearing loss | 5/5 | 5/5 |
| Shortness of breath | 3/5 | 3/5 |
| Symptoms concerning nutrition, metabolism, and development | 5/5 | 3/5 |
| Syncope and collapse | 5/5 | 5/5 |
| Type 2 diabetes | 4/5 | 3/5 |
| Vascular dementia | 5/5 | 3/5 |
| Visual Disturbances | 5/5 | 4/5 |

Manual chart review of the 42 phenotypes initially identified by MHLO validated 33 PASC phenotypes. Rejected phenotypes with less than three out of five confirmed charts in either category are highlighted in grey.

**Table S3.** Multivariate ORs for phenotypes with at least a confidence score above 80 in the cohort or sub-cohorts

| **phenotype** | **model** | **month** | **OR** | **95% C.I.** | **P-value** | **MLHO CS** |
| --- | --- | --- | --- | --- | --- | --- |
| Acute cystitis | 65 and older Male | 6-9 month | 23.3701 | (1.91-336.29) | 0.0145 | 87 |
| Alopecia | all | 3-6 month | 3.0855 | (2.53-3.76) | 0 | 100 |
| Alopecia | 65 and older | 3-6 month | 2.8317 | (1.42-5.49) | 0.0025 | 100 |
| Alopecia | 65 and older Female | 3-6 month | 4.1566 | (2.06-8.55) | 0.0001 | 100 |
| Alopecia | Under 65 | 3-6 month | 3.0098 | (2.39-3.79) | 0 | 100 |
| Alopecia | Under 65 Female | 3-6 month | 2.8741 | (2.24-3.69) | 0 | 100 |
| Alopecia | Under 65 Male | 3-6 month | 1.8587 | (1.05-3.23) | 0.0296 | 81 |
| Anemia during pregnancy | Under 65 Female | 3-6 month | 1.5545 | (1.01-2.39) | 0.0449 | 96 |
| Anemia of chronic disease | 65 and older Female | 6-9 month | 7.7175 | (1.46-40.75) | 0.0147 | 90 |
| Anosmia and dysgeusia | all | 3-6 month | 2.5953 | (1.94-3.46) | 0 | 100 |
| Anosmia and dysgeusia | all | 6-9 month | 2.1023 | (1.4-3.11) | 0.0002 | 100 |
| Anosmia and dysgeusia | Under 65 | 3-6 month | 2.7229 | (1.96-3.77) | 0 | 100 |
| Anosmia and dysgeusia | Under 65 | 6-9 month | 2.2392 | (1.46-3.4) | 0.0002 | 98 |
| Anosmia and dysgeusia | Under 65 Female | 3-6 month | 2.9924 | (2.02-4.45) | 0 | 100 |
| Anosmia and dysgeusia | Under 65 Female | 6-9 month | 2.753 | (1.68-4.47) | 0 | 99 |
| Chronic fatigue syndrome | all | 3-6 month | 1.6059 | (1.22-2.1) | 0.0006 | 98 |
| Chronic fatigue syndrome | all | 6-9 month | 2.0299 | (1.31-3.11) | 0.0013 | 100 |
| Chronic fatigue syndrome | Under 65 | 3-6 month | 1.6573 | (1.21-2.26) | 0.0014 | 99 |
| Chronic fatigue syndrome | Under 65 | 6-9 month | 2.5095 | (1.58-3.93) | 0.0001 | 100 |
| Chronic fatigue syndrome | Under 65 Female | 3-6 month | 1.6137 | (1.13-2.29) | 0.0074 | 99 |
| Chronic fatigue syndrome | Under 65 Female | 6-9 month | 2.4859 | (1.51-4.05) | 0.0003 | 100 |
| Chronic kidney disease, Stage I or II | 65 and older | 3-6 month | 2.194 | (1-4.63) | 0.0432 | 93 |
| Chronic kidney disease, Stage I or II | 65 and older Female | 3-6 month | 3.2231 | (1.26-8.22) | 0.0138 | 90 |
| Conjunctivitis, infectious | Under 65 Male | 3-6 month | 1.9868 | (1.2-3.25) | 0.0068 | 89 |
| Dementias | 65 and older Female | 3-6 month | 2.6686 | (1.22-5.83) | 0.0135 | 83 |
| Diseases of nail, NOS | Under 65 Male | 3-6 month | 2.4611 | (1.3-4.59) | 0.0048 | 87 |
| Disorders of conjunctiva | Under 65 Male | 6-9 month | 4.8208 | (1.34-16) | 0.012 | 91 |
| Dizziness and Light-headedness | 65 and older Female | 6-9 month | 3.628 | (0.94-13.82) | 0.0523 | 92 |
| Heart failure with preserved EF | 65 and older | 3-6 month | 1.8337 | (0.95-3.4) | 0.0613 | 91 |
| Hypertensive heart disease | 65 and older Male | 6-9 month | 18.0705 | (1.83-231.75) | 0.0164 | 88 |
| Iron def. anemias, unspecified | all | 6-9 month | 1.5624 | (1.09-2.22) | 0.0143 | 89 |
| Iron def. anemias, unspecified | Under 65 | 6-9 month | 2.0175 | (1.37-2.95) | 0.0003 | 100 |
| Iron def. anemias, unspecified | Under 65 Female | 3-6 month | 1.2277 | (0.9-1.66) | 0.186 | 87 |
| Iron def. anemias, unspecified | Under 65 Female | 6-9 month | 2.1064 | (1.4-3.15) | 0.0003 | 100 |
| Irregular menstrual cycle | all | 3-6 month | 1.3367 | (1.03-1.74) | 0.0305 | 88 |
| Irregular menstrual cycle | all | 6-9 month | 1.5725 | (1.04-2.36) | 0.0301 | 92 |
| Irregular menstrual cycle | Under 65 | 3-6 month | 1.4097 | (1.06-1.86) | 0.0159 | 90 |
| Irregular menstrual cycle | Under 65 | 6-9 month | 1.7305 | (1.15-2.57) | 0.0072 | 86 |
| Irregular menstrual cycle | Under 65 Female | 3-6 month | 1.1914 | (0.9-1.58) | 0.2247 | 91 |
| Major depressive disorder | Under 65 Female | 6-9 month | 1.5036 | (1.07-2.1) | 0.0179 | 84 |
| Malposition and malpresentation of fetus or obstruction | Under 65 Female | 3-6 month | 1.5905 | (0.97-2.59) | 0.0628 | 86 |
| Neurological disorders | Under 65 | 3-6 month | 1.6268 | (1.16-2.27) | 0.0043 | 95 |
| Neurological disorders | Under 65 Male | 3-6 month | 1.9837 | (1.15-3.4) | 0.0132 | 83 |
| Nonspecific chest pain | all | 3-6 month | 1.2692 | (1.09-1.48) | 0.0021 | 100 |
| Nonspecific chest pain | Under 65 | 3-6 month | 1.2962 | (1.08-1.55) | 0.0048 | 100 |
| Nonspecific chest pain | Under 65 Female | 3-6 month | 1.0726 | (0.87-1.32) | 0.5159 | 92 |
| Nonspecific chest pain | Under 65 Male | 3-6 month | 1.5856 | (1.16-2.17) | 0.0041 | 100 |
| Pain, swelling or discharge of eye | Under 65 Female | 3-6 month | 1.5133 | (0.93-2.44) | 0.0909 | 84 |
| Palpitations | all | 3-6 month | 1.2773 | (1.07-1.53) | 0.0076 | 95 |
| Palpitations | Under 65 | 3-6 month | 1.3391 | (1.09-1.65) | 0.0059 | 100 |
| Palpitations | Under 65 Female | 3-6 month | 1.2041 | (0.95-1.52) | 0.1228 | 100 |
| Peripheral or central vertigo | Under 65 | 6-9 month | 2.207 | (1.23-3.88) | 0.0067 | 82 |
| Pneumonia | all | 3-6 month | 1.6647 | (1.28-2.16) | 0.0001 | 99 |
| Pneumonia | 65 and older | 3-6 month | 1.9205 | (1.03-3.46) | 0.0345 | 99 |
| Pneumonia | 65 and older Female | 3-6 month | 2.2003 | (1.09-4.45) | 0.0265 | 88 |
| Pneumonia | 65 and older Male | 3-6 month | 5.7473 | (0.73-32.38) | 0.058 | 83 |
| Pneumonia | 65 and older Male | 6-9 month | 20.2422 | (2-246.21) | 0.0114 | 89 |
| Pneumonia | Under 65 Male | 3-6 month | 1.8755 | (1.14-3.07) | 0.0128 | 84 |
| Proteinuria | Under 65 Male | 3-6 month | 3.1904 | (1.72-5.96) | 0.0002 | 100 |
| Rash and other nonspecific skin eruption | Under 65 Male | 3-6 month | 1.6029 | (1.12-2.28) | 0.0094 | 96 |
| Sensorineural hearing loss | 65 and older Female | 6-9 month | 3.7484 | (0.97-14.4) | 0.0482 | 91 |
| Shortness of breath | all | 3-6 month | 1.4147 | (1.22-1.64) | 0 | 100 |
| Shortness of breath | all | 6-9 month | 1.4546 | (1.09-1.93) | 0.01 | 96 |
| Shortness of breath | 65 and older Female | 3-6 month | 1.7113 | (0.91-3.25) | 0.0949 | 89 |
| Shortness of breath | Under 65 | 3-6 month | 1.5428 | (1.28-1.85) | 0 | 100 |
| Shortness of breath | Under 65 | 6-9 month | 1.6427 | (1.23-2.18) | 0.0008 | 97 |
| Shortness of breath | Under 65 Female | 3-6 month | 1.3706 | (1.1-1.7) | 0.0044 | 100 |
| Shortness of breath | Under 65 Female | 6-9 month | 1.7222 | (1.22-2.42) | 0.002 | 98 |
| Shortness of breath | Under 65 Male | 3-6 month | 1.6478 | (1.2-2.25) | 0.0017 | 100 |
| Symptoms concerning nutrition, metabolism, and development | 65 and older Female | 3-6 month | 1.9321 | (0.96-3.92) | 0.0645 | 83 |
| Syncope and collapse | Under 65 Male | 6-9 month | 4.7973 | (1.56-13.39) | 0.0037 | 99 |
| Type 2 diabetes | all | 3-6 month | 1.4844 | (1.19-1.85) | 0.0005 | 98 |
| Type 2 diabetes | all | 6-9 month | 1.6505 | (1.12-2.41) | 0.0109 | 81 |
| Type 2 diabetes | Under 65 | 3-6 month | 1.5694 | (1.19-2.06) | 0.0013 | 100 |
| Type 2 diabetes | Under 65 Male | 3-6 month | 2.3437 | (1.53-3.58) | 0.0001 | 100 |
| Vascular dementia | 65 and older Female | 6-9 month | 7.5012 | (1.45-39.04) | 0.0151 | 91 |
| Visual disturbances | 65 and older Male | 6-9 month | 18.4814 | (1.6-265.41) | 0.0225 | 85 |

ORs were calculated using Generalized Linear Models trained on a subset of features identified by MLHO. Confidence Score (CS) are provided for comparison against p values.
